# Differences in Offending Behaviors, Aggression, Substance Use, and Mental Health Problems between Male Drug Dealers and Non-Drug Dealers in Belgian Youth Detention Centers

**DOI:** 10.3390/ijerph192416390

**Published:** 2022-12-07

**Authors:** Athina Bisback, Wouter Vanderplasschen, Olivier F. Colins

**Affiliations:** 1 Department of Special Needs Education at the Faculty of Psychology and Educational Sciences, Ghent University, 9000 Ghent, Belgium; 2 Center for Criminological and Psychosocial Research, Örebro University, SE-701 82 Örebro, Sweden

**Keywords:** adolescent offenders, drug dealing, substance use, violence, mental health

## Abstract

This study investigated whether drug dealing juvenile offenders in Belgium differ from non-drug dealers in levels of violent and non-violent offending behaviors, aggression, substance use, and mental health needs. The current study examined data from 226 16- to 17-year-old male juvenile offenders. Information relating to drug dealing, substance use, and mental health needs were collected through self-report questionnaires. A structured diagnostic interview was used to collect information about past violent and non-violent behaviors. Chi-square tests and multivariate analysis of variance compared non-dealers and dealers and explored if hard-drug dealers and soft-drug dealers differed from each other. Relative to non-drug dealers, drug dealers engaged in more violent offending behaviors, exhibited higher levels of aggression, substance use and oppositional defiant problems, and displayed lower levels of anxiety. Soft- and hard-drug dealers did not differ from each other. To conclude, detained drug dealers are characterized by severe antisocial behavior.

## 1. Introduction

Approximately half of youth in juvenile justice institutions are involved in drug-dealing offenses [1,2]. However, juvenile drug dealing remains an underexplored topic in research that differentiates between types of offenses or offenders [3,4,5,6,7]. The majority of past work classified offenders based on whether they have committed violent or property offenses, or both, but failed to treat drug offenses as a separate type of offense [3,4,5,6,7,8,9,10]. Lai et al. (2016), for example, considered drug use as a nonviolent offense, whereas Colins et al., (2009a) excluded offenders who merely committed drug crimes (i.e., use, possession, and/or dealing of illegal drugs). The few studies that specifically focused on drug crimes as a separate offense type did not differentiate between possession and dealing of illegal drugs [11]. This is unfortunate because individuals who deal drugs differ from individuals who merely possess or use substances in offending history, tendency to act out aggressively, and mental health needs (e.g., [12,13,14,15,16]).

Involvement in drug dealing is regarded a major factor associated with an increase in the likelihood of engaging in violent behaviors [17,18]. Exposure to and participation in systemic violence is dependent on the position one takes within the hierarchy of the drug business, with a higher position being linked to more severe violence [19]. This link can also be observed in gangs. Since roughly three-quarters of all gangs are involved in drug dealing [20], it can be argued that the presence of subcultural hierarchies within the drug dealing business seems to be related to gang membership, at least to some extent. Drug dealers are involved in very specific contexts of subcultural hierarchies (e.g., gangs) that emphasize and reward behaviors of toughness, machismo, and aggression [21]. Men who hold a dominant position may justify their practice to systematically oppress other people (“hegemonic masculinity”) [22]. So, it should come as no surprise that dealers frequently engage in assaultive behavior and intimidation, for example, against other dealers and customers with debts [23], a relationship that was already captured in Goldstein’s systemic model [24]. Engaging in premeditated (or proactive) aggression [25] (to maintain and expand territories and customers [21,23] is not without risk, and may also result in weapon possession to protect oneself during drug dealing activities [20]. Actually, the likelihood of experiencing as well as witnessing violence is high when involved in drug dealing activities [17,18]. Drug dealers might also want to protect themselves against possible new or future attacks, which implies that their aggression can be an automatic and impulsive reaction in response to a perceived threat or frustration (i.e., reactive aggression) [25]. Being a victim of violence may also be a traumatic experience that triggers anger as well as irritability [23], and thus, increases the risk to engage in reactive aggressive behaviors [25]. Clearly, drug dealing activities are associated with an increased likelihood of committing violent offenses and aggressive behaviors. In fact, it is suggested that it is the business of drug dealing that sustains and tolerates the use of violence [26].

There are reasons to suggest that drug dealers and non-drug dealers have unique mental health problems. The exhibition of fear or anxiety may be a weakness incompatible with the macho culture of dealers [21,27], though empirical findings are mixed. To illustrate, Kinner et al., (2009) showed that drug dealers experienced more anxiety and depressive symptoms compared to non-drug dealers [28], but this was not conformed by others [15]. Their involvement in the macho culture of dealing [21] where drug dealers (need to) dominate and oppress others [22], increases the likelihood that these individuals show high levels of oppositional defiant problems. Finally, there is some evidence to suggest that juvenile drug dealers also use more different types of drugs at a high intensity than non-drug dealers [2,13,16]. However, this has to be confirmed in samples of youth who are hallmarked by high levels of (poly)substance use, for example, detained youth [4,29]. Altogether, the exact association between these psychological correlates and drug dealing remain elusive. Filling this void is of clinical importance. Knowledge about mental health problems among subgroups of detained adolescents may enable tailoring of mental health services to the unique needs of these disadvantaged and vulnerable youth [10,30,31]. Mental health problems are in fact dynamic in nature and can be amendable through therapy [15].

In a U.S. juvenile offending population (*N* = 227; 55% boys), it was shown that drug dealers, overall, exhibit higher levels of substance use, and delinquent and other risky behaviors than non-dealers [16]. However, this study also showed that these differences did not consistently emerge across all types of drugs under scrutiny, but in fact varied by the type of drugs being sold (i.e., marihuana, hard drugs, or prescription drugs). However, it was not investigated whether a distinction between hard-drug dealers and soft-drug dealers is of clinical importance. This is unfortunate because the investigation of variations between different types of drug dealers (e.g., soft-drug dealers versus hard-drug dealers) may identify key differences between these types of drug dealers in relation to demographic and psychosocial correlates, delinquency, and to substance use behavior [2]. While the Shook et al., (2011, 2013) studies started to fill this knowledge gap [2,16], it is uncertain to what extent their findings can be generalized to other countries, where the organization of juvenile justice (e.g., prosecution policy), mental health care (e.g., availability of services), and sociodemographic make up (e.g., different ethnic “minority” groups) differs greatly from the U.S. [32].

### Aims of the Study

This study was designed to bolster what is known about drug dealing adolescent boys in Belgian youth detention centers. Specifically, it was tested if drug dealers engage in more violent and non-violent behaviors [16,17,18,19,20], exhibit higher levels of reactive and proactive aggression [17,18,21], substance use [12,13,16], and mental health problems [13,14,28,33] than non-drug dealers. The current study also explored if it bears relevance to differentiate between soft-drug dealers and hard-drug dealers (see the Supplementary Materials for a checklist of items that should be included in reports of cross-sectional studies).

## 2. Materials and Methods

### 2.1. Participants

The present study used data from an ongoing study with delinquent male adolescents. The target population consisted out of all boys aged 16 or 17 residing in two youth detention centers in Belgium (see [34,35] for more information about the detention centers and the relevant legislation). Boys were eligible to participate if they had no problems that could jeopardize their well-being or participation, and had sufficient knowledge of Dutch. Data used in this study were collected between August 2019 and October 2020. Active consent for participation was solicited from 239 youth (see Section 2.2. Procedure). Of the 227 participants that provided consent and enrolled in the study, the parents from one participant asked us to delete the data from their son, resulting in a total sample of 226 boys (M_age_ = 16.95, SD_age_ = 0.59). About 38% of the 226 boys’ parents were both born in Belgium, whereas 17.6% and 39.8% had one or no parent(s) who was born in Belgium.

### 2.2. Procedure

For each 16- or 17-year-old boy in the detention center written consent was asked from his psychologist. Next, if the psychologist consented, the boys were individually approached and received oral information about the study aims. Participants were informed that all the information would be treated confidentially, and that refusal to participate would not affect their judicial status or stay in the institution. A written informed consent was signed by the boy before starting the standardized assessment protocol. Finally, the detained boys’ parents/caretaker received a letter with information about the aims and practical aspects of the study and were informed that they could decline participation (i.e., passive informed consent). Participants were assessed in a private area by two PhD-students (including first author) in three separate blocks to avoid participant fatigue. In these blocks the boys completed questionnaires, performed computerized experiments, and/or were interviewed by means of semi-structured diagnostic interviews. In return for their participation, each participant received a small financial compensation (€25). This study was approved by the institutional review board of Ghent University and the board of the youth detention centers (YDC).

### 2.3. Measures

#### 2.3.1. Drug Dealing

Drug dealing behavior was measured using a questionnaire specifically designed for this study. The participants were asked if they had ever sold drugs (yes or no), how often (frequency), how old they were the first time they sold drugs (age of onset), and which drugs they had sold. A boy was identified as dealer if he indicated that he had ever sold drugs (all the results remained substantially similar when only looking at individuals that sold drugs for a longer period). More strict delineation of drug dealer groups (i.e., never—once—several times—longer period) resulted in only a small number of individuals in the ‘once’ and ‘several times’ group. The distinction between soft- and hard-drugs was based on previous research [16] as well as Belgian legislation [36]. Cannabis (i.e., marijuana and hash) was regarded as soft-drugs, whereas all the others were characterized as hard-drugs.

#### 2.3.2. Violent and Non-Violent Offenses

Violent and non-violent offenses were measured by means of the conduct disorder module of a semi-structured diagnostic interview, the Kiddie Schedule for Affective Disorders and Schizophrenia—Present and Lifetime version (K-SADS-PL) [37,38]. To facilitate scoring of the information derived through the interview, file information was collected as well. In case of discrepancy between different sources, the clinical judgment of the investigator was decisive [38]. In line with DSM-5 [39] we also classified the 15 conduct disorder (CD) symptoms under four broad categories, of which 1 captures violent behavior (i.e., aggression to people and animals; 7 CD symptoms). Three categories capture non-violent behavior being: destruction of property (2 CD symptoms); deceitfulness or theft (3 CD symptoms); and serious violations of rules (3 CD symptoms). For each of these four broad categories we calculated the percentage of boys who committed at least one offense in the past six months and the number of offenses committed by the participants in the last six months. In line with the K-SADS manual, a CD symptom was considered present if the boy received the highest score on a 4-point response scale. Good concurrent validity, interrater agreement and test–retest reliability has been reported for the CD module [37].

#### 2.3.3. Gang Membership

Gang membership was also measured using the CD module of the K-SADS-PL. In line with past work [40,41], gang membership was coded “1” if the participant reported being a gang member before entering the institution, and coded “0” if he did not see himself as a gang member.

#### 2.3.4. Aggression

Aggression was measured via the Reactive-Proactive Questionnaire (RPQ) [25,42]. The RPQ is a self-report questionnaire that includes 23 items that distinguish between reactive and proactive aggression and can be scored on a 3-point scale (0 = never, 1 = sometimes, 2 = often). Eleven items tap into reactive aggression (e.g., reacted angrily when provoked by others), and twelve items are aimed to assess proactive aggression (e.g., had fights with others to show who was on top). The RPQ has been found to be a psychometrically sound measure in criminal justice-involved youth [25,42,43]. For the current study, internal reliability for the Proactive Aggression scale was α = 0.86 and for the Reactive Aggression scale was α = 0.84.

#### 2.3.5. Mental Health Problems

Depressive problems, anxiety problems, attention deficit/hyperactivity problems, and oppositional defiant problems that occurred during the past 6 months were assessed by mean of the corresponding DSM-oriented problem scales of the Dutch Youth Self-Report (YSR) [44]. The items in these scales use a 3-point response scale ranging from 0 (not at all) to 2 (often). The DSM-oriented problem scales have been found to be a psychometrically sound measure in criminal justice-involved youth [45]. In the current study, the YSR had an internal consistency of α = 0.73 for anxiety problems, α = 0.72 for depressive problems, α = 0.72 for attention deficit/hyperactivity problems and α = 0.59 for oppositional defiant problems.

#### 2.3.6. Substance Use

Substance use was measured by asking if participants had ever used one of the following substances: alcohol, marijuana, amphetamines (including speed and ecstasy), cocaine, and other substances (e.g., lysergic acid diethylamide (LSD), magic mushrooms or heroin). For each substance participants also reported how old they were when they first used the substance (age of onset) and how often they had used a particular substance in the year before being sent to the juvenile institution (number of days per week). A boy was classified as ‘user’ of a particular substance if he indicated that he had ever used that specific substance. An extra variable was created measuring polysubstance use, which can be defined as the sum of all the different substance categories, including alcohol, they had ever used in their lifetime (range = 0 to 5).

#### 2.3.7. Sociodemographics

Ethnic origin of the boys was determined on the basis of the nationality of both parents [46]. Specifically, three different groups were created: Belgian origin (both parents had Belgian nationality), non-Belgian origin (both parents had another nationality) or mixed origin (one parent does not have the Belgian nationality). Several questions were posed to obtain a sense of respondents’ socioeconomic status. Degree of parental education was conceptualized by one of three categories: no diploma/primary school diploma, secondary school diploma, or graduate school or university diploma. For the employment variable, a two-level variable was created based on the respondents answer: employed or unemployed.

#### 2.3.8. Statistical Analyses

Descriptive information (Mean, SD and/or percentages) for violent and non-violent behaviors, aggression, substance use and mental health problems among dealers and non-dealers was first calculated. Next, dealers and non-dealers were compared with regards to different features (predictors). In case of a categorical predictor (i.e., violent and non-violent behavior and substance use), chi-square tests were used. To obtain a sense on the strength and direction of this relationship the appropriate measure of association, Cramér’s *V*, was calculated. The interpretation of Cramér’s *V* with one degree of freedom was as follows: 0.10 indicated a small effect size, 0.30 a medium effect size and 0.50 a large effect size [47]. In case of one continuous predictor variable (e.g., polysubstance use), an independent sample *t*-test was used. When dealing with related continuous predictor variables (e.g., four offense categories, reactive and proactive aggression and DSM-oriented problem scales), a Multivariate Analysis of Variance (MANOVA) was used. Although the samples sizes were not equal among groups, it was permitted to perform this analysis as we were not able to reject the null hypothesis of population normality by means of Kolmogorov–Smirnov test of normality, and homogeneity of variances was assumed by Levene’s Test. If the multivariate test was significant, follow-up tests (univariate ANOVAs) were performed to determine the exact differences between dealers and non-dealers. Cohen’s *d* effect sizes were calculated to describe the results in terms of measures of magnitude with 0.20 as a small effect, 0.50 as a medium effect and 0.80 as a large effect [47]. Finally, to explore if there were differences between soft-drug dealers, hard-drug dealers, and non-dealers a similar strategy was implemented with the extension of the investigation of pairwise comparisons as well as Bonferroni post hoc tests were performed. An alpha level of *p* < 0.05 was used as indicator of statistical significance. All analyses were conducted with the use of IBM SPSS Statistics 26.

## 3. Results

### 3.1. Dealers versus Non-Dealers

#### 3.1.1. Sociodemographic Features

The sample consisted of 79 (34.9%) non-dealers and 147 (65.0%) dealers. The groups did not differ with regards to educational degree of father [*X*^2^(2, *N* = 98) = 0.46, *p* = 0.80], educational degree of mother [*X*^2^(2, *N* = 150) = 1.07, *p* = 0.59], employment of mother [*X*^2^(1, *N* = 166) = 1.10, *p* = 0.30], and origin [*X*^2^(2, *N* = 217) = 0.004, *p* = 1.0]. However, non-dealers more often had unemployed fathers than drug dealers [*X*^2^(1, *N* = 128) = 7.56, *p* = 0.006].

#### 3.1.2. Violent and Non-Violent Offenses

Dealers engaged in more violent behaviors [*F*(1, 223) = 25.77, *p* < 0.001, Cohen’s *d* = 0.71], made more use of deceitfulness or engaged in theft [*F*(1, 223) = 20.92, *p* < 0.001, Cohen’s *d* = 0.64], and showed more serious violations of rules [*F*(1, 223) = 12.65, *p* < 0.001, Cohen’s *d* = 0.49], see Table 1. More specifically, dealers more often bullied, threatened or intimidated others, made more use of weapons, had stolen more while confronting victims, more often initiated physical fights, were more likely to have a history of breaking and entering, stealing without confrontation, running away from home and staying out at night without parental permission (with small to medium effect sizes, ES). Dealers were more likely to be a gang member than non-dealers [49.7% versus 30.4%; *X*^2^(1, *N* = 225) = 7.42, *p* < 0.01], though the ES was small (i.e., Cramér’s *V* = 0.18).

#### 3.1.3. Aggression

Table 2 reports group differences in aggression. The multivariate test resulted in a statistically significant difference between dealers and non-dealers, *F*(2, 220) = 17.98, *p* < 0.001, Wilks’ Λ = 0.86, partial *η*^2^ = 0.14. As shown in Table 2, dealers exhibited higher levels of reactive aggression (medium ES) and proactive aggression (large ES).

#### 3.1.4. Substance Use

Overall, dealers reported significantly more substance use compared to non-dealers for all different substances (medium ES; see Table 3). On average, dealers have tried between two and three types of substances. Non-dealers have ever tried between one and two types of substances. An independent-samples *t*-test indicated a significant difference in polysubstance use between non-dealers (M = 1.65, SD = 1.00) and dealers (M = 2.74, SD = 1.22); *t*(211.43) = −7.25, *p* < 0.001, Cohen’s *d* = 0.95).

#### 3.1.5. Mental Health Problems

The multivariate test indicated a statistically significant difference between dealers and non-dealers, *F*(4, 221) = 7.71, *p* < 0.001, Wilks’ Λ = 0.88 and partial *η*^2^ = 0.12. Separate univariate ANOVAs on the outcome variables (see Table 4) revealed that dealing had a significant effect on both anxiety problems and oppositional defiant problems, with dealers experiencing significantly less anxiety and more oppositional defiant problems, with small to medium effect sizes. The groups did not differ in terms of depressive problems and attention deficit/hyperactivity problems.

### 3.2. Exploratory Analyses of Soft-Drug Dealers, Hard-Drug Dealers, and Non-Dealers

#### 3.2.1. Sociodemographic Features

The sample consisted of 79 (34.9%) non-dealers 63 (27.8%) soft-drug dealers and 83 (36.7%) hard-drug dealers. The groups did not differ with regards to educational degree of father [*X*^2^(4, *N* = 97) = 0.68, *p* = 0.95], educational degree of mother [*X*^2^(4, *N* = 149) = 3.96, *p* = 0.41], employment of mother [*X*^2^(2, *N* = 165) = 1.42, *p* = 0.49], and origin [*X*^2^(4, *N* = 216) = 0.61, *p* = 0.96]. Non-dealers more often had unemployed fathers than both groups of drug dealers [*X*^2^(2, *N* = 127) = 7.45, *p* = 0.02].

#### 3.2.2. Violent and Non-Violent Offenses

The multivariate test indicated a statistically significant difference between non-dealers, soft-drug dealers and hard-drug dealers, [*F*(8, 436) = 5.09, *p* < 0.001, Wilks’ Λ = 0.84, partial *η*^2^ = 0.09]. Similar to the initial results of non-dealers versus dealers, the categories of aggression to people and animals [*F*(2, 221) = 14.13, *p* < 0.001, Cohen’s *d* = 0.87], deceitfulness or theft [*F*(2, 221) = 10.30, *p* < 0.001, Cohen’s *d* = 0.75], and serious violations of rules [*F*(2, 221) = 6.28, *p* < 0.001, Cohen’s *d* = 0.59] were statistically significantly different for the three groups. In addition, soft-drug dealers and hard-drug dealers did not differ in terms of bullying, weapon use, robbery, burglary, theft and running away from home but did so more compared to non-dealers. However, hard-drug dealers were more physically cruel to people compared to soft-drug dealers and non-dealers (small ES), and were more likely to be a gang member (small ES; see Table 5).

#### 3.2.3. Aggression

There was a statistically significant difference according to the multivariate test between non-dealers, soft-drug dealers and hard-drug dealers, [*F*(4, 436) = 9.35, *p* < 0.001, Wilks’ Λ = 0.85, partial *η*^2^ = 0.08], see Table 6. There are differences between the three groups in reactive aggression [*F*(2, 219) = 12.61, *p* < 0.001, partial *η*^2^ = 0.10] and proactive aggression [*F*(2, 219) = 17.86, *p* < 0.001, partial *η*^2^ = 0.14], with large ES. Pairwise comparisons revealed that reactive aggression was significantly lower for non-dealers compared to soft-drug dealers (−2.32, *p* = 0.01) and hard-drug dealers (−3.60, *p* < 0.001). For proactive aggression, non-dealers showed lower aggression levels compared to soft- (−3.32, *p* < 0.001) and hard-drug dealers (−4.71, *p* < 0.001). There was no statistically significant difference between the soft-drug dealers and hard-drug dealers in reactive aggression (*p* = 0.29) nor proactive aggression (*p* = 0.31).

#### 3.2.4. Substance Use

Overall, chi-square results can be found in Table 7. For alcohol, cannabis, amphetamines and cocaine, non-dealers used significantly less substances compared to both soft-drug dealers and hard-drug dealers. Soft-drug dealers and hard-drug dealers showed similar habits in the use of alcohol, cannabis and cocaine. Usages of ‘other types of drugs’ was similar between non-dealers and soft-dealers, but differences arose between non-dealers and hard-drug dealers, and soft-drug dealers and hard-drug dealers. There was a significant difference in polysubstance use for non-dealers (M = 1.65, SD = 1.00), soft-drug dealers (M = 2.46, SD = 0.98) and hard-drug dealers (M = 2.96, SD = 1.35); *F*(2, 222) = 27.58, *p* < 0.001. Post hoc test revealed that hard-drug dealers are significantly more likely to have used more types of drugs followed by soft-drug dealers and non-dealers.

#### 3.2.5. Mental Health Problems

There was a statistically significant difference between non-dealers, soft-drug dealers and hard-drug dealers shown by the multivariate test [*F*(8, 438) = 3.92, *p* < 0.001, Wilks’ *Λ* = 0.87, partial *η*^2^ = 0.07], see Table 8. This difference is due to the oppositional defiant problems scale [*F*(2, 222) = 7.77, *p* = 0.001, partial *η*^2^ = 0.07] and the anxiety problems scale [*F*(2, 222) = 3.74, *p* = 0.03, partial *η*^2^ = 0.03], with a small to medium effect size. For the oppositional defiant problem scale, pairwise comparisons indicated that there are statistically significant differences between non-dealers and hard-drug dealers (−1.31, *p* < 0.001). Specifically, non-dealers experienced less oppositional defiant problems compared to hard-drug dealers, whereas non-dealers and soft-drug dealers only differed in anxiety (+1.31, *p* = 0.04).

## 4. Discussion

This is one of the first studies to scrutinize differences between adolescent dealers and non-dealers in youth detention centers in a non-U.S. context. Notwithstanding that there are several differences between youth who engage in drug dealing and those who do not, the most important finding is that detained dealers in Flemish Belgium constitute a more severe group of offenders, a finding that corroborates with past work with U.S. criminal justice involved youth [16]. Overall, dealers engaged in more violent and more offending behaviors, had higher aggression scores and presented more oppositional defiant problems compared to non-dealers. In the following part, we will reflect upon the most important findings.

### 4.1. Offenses and Aggression

Dealers are a seriously violent and dangerous group as shown by heightened levels of weapon use, intimidation, initiating fights, violent robbery and proactive aggression. Consistent with previous research, dealers are also more likely to be a gang member [16], which can be explained by the aforementioned (see Section 1. Introduction) concepts of hegemonic masculinity [22] and macho behavior [20,21]. Elevated proactive aggression levels might be explained by the systemic model [24]. Premeditated aggressive behaviors can take place when ‘settlements’ are required with customers with debts as well as when other dealers are crossing the premises. These heightened proactive levels may also be a function of social learning [48], with on the one hand the idea that proactive aggression pays off and gets them what they want, whereas on the other hand individuals engaging in proactive aggression are more easily attracted by the business because this behavior is rewarded and appreciated in this context. Our results urge that this group should be closely monitored during detention as well as after release because recidivism rates for adult dealers are high [49]. Dealers are also more likely to engage in reactive aggression, and thus, act in the spur of the moment without taking the consequences into account. This urges for screening of the different functions of aggression rather early in the admission process [43].

One type of offense that has similar rates among dealers and non-dealers is the destruction of property. This can be partly explained by Goldstein’s (1985) economic-compulsive model that suggests that drug dealers may need more money than other offenders to provide for their own habit of drug use or more broadly their lifestyle, and that offending without ‘actual rewards’ such as the destruction of one’s property will not benefit them enough [24]. One may even say that when an individual is involved in the drug dealing business, the engagement in violent behaviors is an actual reward in itself (e.g., because others will praise this tough appearance). Goldstein’s (1985) systemic model suggests that by engaging in violent behavior one posits himself as someone that cannot be messed with [21], resulting in other people, such as customers being scared or intimidated resulting in paying their debts in time [24]. For the dealer, this is a success in relation to his hegemonic masculinity, which will only strengthen this behavior over time [22].

As expected, drug dealers engaged in more violent behaviors, made more use of deceitfulness or engaged in theft, and showed more serious violations of rules. Several explanations can be postulated to explain these findings. First, general versatility of crime among detainees is the rule, rather than the exception [23,50]. The even higher versatility for drug dealers can be explained by the fact that drug dealers may present generally higher levels of risk-taking behavior and thus engage in more different types of offending behaviors [2,16,51,52].

### 4.2. Substance Use

Dealers used more alcohol, marijuana, amphetamines, cocaine and other drugs relative to non-dealers. This finding can be explained by the economic-compulsive model stating that substance users may deal drugs because it provides them with an income to maintain their own drug use [24]. Hence, the roles of buyer and seller are therefore not fixed ones [23]. Dealers have easy access to very different substances making the usage of a new substance more common, and their risk-taking behavior [2,16,51] may trigger them to trying out more types of drugs. Furthermore, substance use was also high for non-dealers, which is in line with the earlier observation that substance use among criminal justice involved male adolescents is highly prevalent [16,29]. Our results highlight the relevance of implementing a screening instrument of substance use upon entry as well as the remainder of their stay, because juveniles often use substances within the institution as well as outside when granted opportunities to leave for several hours. More importantly, substance use also increases criminal recidivism risk, including the engagement in drug-related offenses after release to society [53]. Therefore, it is important for future work to take this into account in the context of risk assessments as well as prognosis. Prevention and treatment for substance use may aid in the reduction in someone’s involvement in the drug dealing business.

### 4.3. Mental Health Problems

Mental health problems differed between dealers and non-dealers. Contrary to earlier research [16], dealers did not experience more anxiety compared to non-drug dealers [28]. In this study, dealers experienced less anxiety and engaged in more oppositional defiant behaviors. Both can be understood again by means of the hegemonic masculinity concept [22]. Tough and macho behaviors are rewarded within the drug hierarchy [21]. Controlling others is justified [22]. One might even suggest that anxiety will not be tolerated and does not aid in the survival in this specific business and can be regarded as a risk factor for failing. Moreover, being called oppositional defiant can work rather reinforcing as tough behaviors are wanted within this business. This is in line with the finding that hard-drug dealers showed more severe oppositional problems compared to non-dealers. More importantly, one could speculate that within the hierarchy of the dealing business, a psychopath-like profile is to be expected. Individuals with psychopathy show an absence of anxiety, on the contrary they show elevated levels of oppositional behaviors as well as proactive aggression [54], and thus, are likely to thrive in the context of dealing. In fact, preliminary evidence suggests that psychopathic individuals can easily influence others to use substances and are more likely to actually have already been involved in dealing [55]. These findings underscore the need to screen for specific mental health behaviors [56] and psychopathic traits [57] when arriving in the juvenile institution.

### 4.4. Distinction between Soft-Drug Dealers and Hard-Drug Dealers

Contrary to our expectations and previous findings [2,16], differentiating between soft-drug dealers and hard-drug dealers does not seem to be of utmost relevance. This may indicate that it is rather the involvement in the business per se and not the type of drugs being sold that is of importance in explaining the relationship with violence [26].

Shook et al. (2013) assigned U.S. non-institutionalized adolescents who reported past-year drug selling into three mutually exclusive groups based on demographics, substance use, behavioral and psychosocial characteristics. Three latent classes were identified: dabblers, delinquents and externalizers. Dabblers showed low levels of violence as well as elevated levels of alcohol and cannabis use; delinquents were characterized by high levels of violence, moderate levels of alcohol and cannabis use, and low levels of illicit drug use; and externalizers showed heightened levels of violent and delinquent behaviors as well as (illicit) drug use [2]. In our study, hard-drug dealers used more assaultive behaviors. One might argue that from all the offenses included in this study, assault is in fact the most severe form of physical violence. Hard-drug dealers were also more likely to have used other types of drugs such as LSD and heroin compared to soft-drug dealers and non-dealers, and used more different types of drugs followed by soft-drug dealers and non-dealers, a finding that corroborates with past research [16]. We can thus tentatively argue that the participants in this study comprise a mixture of both ‘delinquents’ and ‘externalizers’, with soft-drug dealers more likely to fit into the category of delinquents, whereas the heightened levels of own substance use placing hard-drug dealers into the externalizers group. The dabblers are likely not represented in this study, as only the most severe or violent youth are being sent to a juvenile institution. One notable difference between externalizers and our hard-drug dealers is that membership of the externalizers group was associated with an increased likelihood of having received a lifetime anxiety diagnosis. This difference can be explained by methodological differences in examining psychological wellbeing. Shook et al., (2013) determined the presence of a diagnosis of anxiety upon whether the individuals were ever informed by a doctor that they met these criteria [2], whereas we relied upon a standardized questionnaire to ask about anxiety symptoms in the last six months.

### 4.5. Recommendations for Clinical Practice

This study indicates that there are in fact differences in psychosocial functioning between juvenile drug dealers and non-dealers. Unfortunately, the juvenile courts are not always aware of the youngster being involved in dealing behaviors. Many juvenile offenders are, in fact, involved in drug dealing behaviors without knowledge of the juvenile court. Practitioners should, therefore, keep in mind that the offense for which a youngster is referred to the institution is not necessarily the only offense that a person committed. Moreover, this study indicates that a rather high percentage of youth involved in juvenile justice is also involved in drug dealing behaviors, and that dealers engage in more violent and more offending behaviors, have higher aggression scores and present more oppositional defiant problems compared to non-dealers. It is important for clinical practitioners to develop a trust relationship with the youngster so that he will also be open about other committed criminal behaviors, such as dealing and the needs that go along with it. Tertiary prevention programs for dealers or users should focus on the reasons why someone engages in dealing behaviors or uses a specific drug. Every individual strives towards achieving primary goods that are innately satisfying and fundamental to their well-being (i.e., Good Lives Model). When working with detained youth these primary goods can best be represented by concrete goals reflecting satisfaction of the offender’s own needs and what’s in it for them [58,59]. This can be particularly important for dealers and users who are referred to mandatory treatment settings, such as the youth detention centers in the current study. Returning agency, giving control back and supporting autonomy by working together to fulfill a juvenile’s own life priorities may make the youngster more motivated to actively engage in therapy [60] and work towards finding other ways besides dealing (or committing offenses) to satisfy their primary goods.

### 4.6. Limitations and Future Research

As always, findings must be interpreted in light of several limitations. First, substance use and dealing were not measured by means of a well-validated self-report scale. However, studies have indicated that adolescents’ own accounts about drug dealing and substance use can be used as valid measurements [12]. Second, with one notable exception (i.e., file information used to check reliability of provided answers on the K-SADS-PL), we solely relied on youth self-report, thereby increasing the risk for untruthful answers and inflated correlations due to common-method bias [61]. Third, the distinction between soft- and hard-drugs was based on previous research as well as Belgian legislation that distinguishes between cannabis use and other illegal drugs among adults. Cannabis (i.e., marijuana and hash) was, thus, regarded as soft-drugs, whereas all the others were characterized as hard-drugs. However, dividing drugs into categories is not obvious and differs across countries: there is no internationally universal accepted distinction present at this time [62]. Fourth, we solely relied on data from adolescent boys. Replication in other samples is warranted, in particular in female samples in which the concept of hegemonic masculinity is rather problematic and cannot be applied as a reason to engage in certain violent behaviors. Female and male young offenders do seem to be a different type of offender and have unique roles as dealers [63]. Fifth, participants were identified as ‘user’ of a particular substance if they had indicated that they had ever used that specific substance. This is a very low threshold, especially for cannabis and alcohol. Future research should take into account the frequency with which these substances are used. Furthermore, in this study we measured proactive and reactive aggression, but it is impossible to know if participants exhibited these functions of aggression during drug dealing activities. Finally, the cross-sectional study design does not allow to determine whether, for example, engaging in violent behaviors or substance use preceded or succeeded drug dealing. A longitudinal design will be more suitable to first investigate differences in criminal recidivism between the groups, and second to confirm the exact prospective associations between the different variables and drug dealing.

## 5. Conclusions

First and foremost, in this research dealers were more violent offenders and engaged in more different types of aggressive behaviors, both reactive as well as proactive aggression. There appears to be no added value to make an extra distinction between soft-drug dealers and hard-drug dealers. Furthermore, there is a rather large association between being a seller as well as a user, with sellers using more different types of drugs.

## Figures and Tables

**Table 1:** Violent and Nonviolent Offenses Among Non-Dealers (*N* = 78) and Dealers (*N* = 147).

	Non-Dealers *N* (%)	Dealers *N* (%)	χ^2^	Cramer’s *V*
Aggression to people and animals—violent offending category	73 (92.4)	143 (97.3)	26.39 ***	0.34
Often bullies, threatens, or intimidate others	50 (63.3)	125 (85.0)	12.92 ***	0.24
Often initiates physical fights	54 (68.4)	119 (81.0)	3.94 *	0.13
Has used a weapon that can cause serious physical harm to others	10 (12.7)	63 (42.9)	20.98 ***	0.31
Has been physically cruel to people	38 (48.1)	86 (58.5)	1.97	0.09
Has been physically cruel to animals	1 (1.3)	4 (2.7)	0.49	0.05
Has stolen while confronting a victim	32 (40.5)	105 (71.4)	19.78 ***	0.30
Has forces someone into sexual activity	7 (8.9)	6 (4.1)	2.24	0.10
Destruction of property	35 (44.3)	72 (49.0)	0.70	0.06
Has deliberately engaged in fire setting with the intention of causing serious harm	8 (10.1)	13 (8.8)	0.12	0.02
Has deliberately destroyed others’ property	30 (38.0)	68 (46.3)	1.26	0.08
Deceitfulness or theft	57 (72.1)	132 (89.8)	21.69 ***	0.31
Has broken into someone else’s house, building or car	23 (29.1)	77 (52.4)	10.82 **	0.22
Often lies to obtain goods or favors or to avoid obligations	40 (50.6)	86 (58.5)	1.08	0.07
Has stolen items of nontrivial value without confronting a victim	33 (41.8)	109 (74.1)	22.19 ***	0.31
Serious violations of rules	30 (37.9)	91 (61.9)	12.67 **	0.24
Often stays out at night despite parental prohibitions, beginning before age 13	1 (1.3)	13 (8.8)	4.99 *	0.15
Has run away from home overnight	28 (35.4)	82 (55.8)	8.06 **	0.19
Is often truant from school, beginning before age 13 years	6 (7.6)	23 (15.6)	2.87	0.11

Note. Cramer’s *V* of 0.10 is indicative of a small effect size, 0.30 of a medium effect size and 0.50 of a large effect size. * *p* < 0.05; ** *p* < 0.01; *** *p* < 0.001.

**Table 2:** Reactive and Proactive Aggression Used by Non-Dealers *(N* = 79) and Dealers (*N* = 147).

	Non-Dealers	Dealers		
	M (SD)	M (SD)	*F*(1, 221)	Cohen’s *d*
Reactive aggression	10.73 (5.05)	13.79 (4.31)	22.61 ***	0.67
Proactive aggression	6.38 (4.85)	10.55 (5.25)	33.55 ***	0.82

Note. Cohen’s *d* of 0.20 is indicative of a small effect, 0.50 of a medium effect and 0.80 of a large effect. *** *p* < 0.001.

**Table 3:** Substance Use Among Dealers (*N* = 79) and Non-Dealers (*N* = 147).

	Non-Dealers	Dealers		
	*N* (%)	*N* (%)	χ^2^	Cramer’s *V*
Alcohol	63 (79.7)	143 (97.3)	19.58 ***	0.29
Marijuana	51 (64.6)	138 (93.9)	32.27 ***	0.38
Amphetamines (speed, ecstasy)	9 (11.4)	54 (36.7)	16.41 ***	0.27
Cocaine	6 (7.6)	42 (28.6)	13.52 ***	0.25
Other (LSD, mushrooms, heroin)	1 (1.3)	27 (18.4)	13.85 ***	0.25

Note. Cramer’s *V* of 0.10 is indicative of a small effect size, 0.30 of a medium effect size and 0.50 of a large effect size. *** *p* < 0.001.

**Table 4:** Mental Health Problems Among Non-Dealers (*N* = 79) and Dealers (*N* = 147).

	Dealers	Non-Dealers		
	M (SD)	M (SD)	*F*(1, 222)	Cohen’s *d*
Anxiety problems	4.86 (3.43)	3.69 (2.85)	7.54 **	0.38
Depressive problems	6.68 (4.55)	5.90 (4.00)	1.80	0.19
Attention deficit/hyperactivity	5.54 (2.69)	5.74 (3.07)	0.23	0.07
Oppositional defiant problems	3.53 (2.15)	4.65 (2.13)	14.00 ***	0.52

Note. Cohen’s *d* of 0.20 is indicative of a small effect, 0.50 of a medium effect and 0.80 of a large effect. ** *p* < 0.01; *** *p* < 0.001.

**Table 5:** Violent and Nonviolent Offenses Among Non-Dealers (*N* = 79), Soft-Drug Dealers (*N* = 63) and Hard-Drug Dealers (*N* = 83).

	Non-Dealers *N* (%)	Soft-Dealers *N* (%)	Hard-Dealers *N* (%)	χ^2^
Aggression to people and animals—violent offending category				
Often bullies, threatens or intimidate others	50 (63.3) ^ab^	51 (81.0) ^a^	73 (88.0) ^b^	13.73 **
Often initiates physical fights	54 (68.4)	50 (79.4)	68 (81.9)	3.96
Has used a weapon that can cause serious physical harm to others	10 (12.7) ^ab^	23 (36.5) ^a^	39 (47.0) ^b^	22.29 ***
Has been physically cruel to people	38 (48.1) ^b^	30 (47.6) ^c^	55 (66.3) ^bc^	6.88 *
Has been physically cruel to animals	1 (1.3)	1 (1.6)	3 (3.6)	1.17
Has stolen while confronting a victim	32 (40.5) ^ab^	46 (73.0) ^a^	58 (69.9) ^b^	19.60 ***
Has forces someone into sexual activity	7 (8.9)	2 (3.2)	4 (4.8)	2.38
Destruction of property—property offending category				
Has deliberately engaged in fire setting with the intention of causing serious harm	8 (10.1)	6 (9.5)	7 (8.4)	0.16
Has deliberately destroyed others’ property	30 (38.0)	30 (47.6)	38 (45.8)	1.41
Deceitfulness or theft				
Has broken into someone else’s house, building or car	23 (29.1) ^ab^	33 (52.4) ^a^	43 (51.8) ^b^	10.50 **
Often lies to obtain goods or favors or to avoid obligations	40 (50.6)	32 (50.8)	53 (63.9)	3.47
Has stolen items of nontrivial value without confronting a victim	33 (41.8) ^ab^	48 (76.2) ^a^	60 (72.3) ^b^	22.09 ***
Serious violations of rules				
Often stays out at night despite parental prohibitions, beginning before age 13	1 (1.3)	5 (7.9)	8 (9.6)	5.22
Has run away from home overnight	28 (35.4) ^ab^	36 (57.1) ^a^	45 (54.2) ^b^	7.93 *
Is often truant from school, beginning before age 13 years	6 (7.6)	11 (17.5)	12 (14.5)	3.22

Note. * *p* < 0.05; ** *p* < 0.01; *** *p* < 0.001 ^a^ non-dealers versus soft-drug dealers; ^b^ non-dealers versus hard-drug dealers; ^c^ soft-drug dealers versus hard-drug dealers.

**Table 6:** Reactive and Proactive Aggression used by Non-Dealers (*N* = 79), Soft-Drug Dealers (*N* = 63) and Hard-Drug Dealers (*N* = 83).

	Non-Dealers	Dealers			
		Soft-Drugs	Hard-Drugs		
	M (SD)	M (SD)	M (SD)	*F*(2, 219)	Cohen’s *d*
Reactive aggression	10.73 (5.05) ^ab^	13.05 (4.39) ^a^	14.33 (4.21) ^b^	12.61 ***	0.83
Proactive aggression	6.38 (4.85) ^ab^	9.69 (5.32) ^a^	11.08 (5.09) ^b^	17.86 ***	0.99

Note. Cohen’s *d* of 0.20 is indicative of a small effect, 0.50 of a medium effect and 0.80 of a large effect. *** p < 0.001 ^a^ non-dealers versus soft-drug dealers; ^b^ non-dealers versus hard-drug dealers.

**Table 7:** Substance Use Among Non-Dealers (*N* = 79), Soft-Drug Dealers (*N* = 63) and Hard-Drug Dealers (*N* = 83).

	Non-Dealers	Dealers			
	*N* (%)	Soft *N* (%)	Hard *N* (%)	χ^2^	Cramer’s *V*
Alcohol	63 (79.7) ^ab^	61 (96.8) ^a^	81 (97.6) ^b^	19.44 ***	0.29 ***
Marijuana	51 (64.6) ^ab^	59 (93.7) ^a^	78 (94.0) ^b^	31.99 ***	0.38 ***
Amphetamines	9 (11.4) ^ab^	17 (27.0) ^a^	37 ^a^ (45.1) ^b^	22.70 ***	0.32 ***
Cocaine	6 (7.6) ^ab^	14 (22.2) ^a^	28 (33.7) ^b^	16.52 ***	0.27 ***
Other	1 (1.3) ^b^	4 (6.3) ^c^	23 (27.7) ^bc^	28.96 ***	0.36 ***

Note. Cramer’s *V* of 0.10 is indicative of a small effect size, 0.30 of a medium effect size and 0.50 of a large effect size. *** *p* < 0.001. ^a^ non-dealers versus soft-drug dealers; ^b^ non-dealers versus hard-drug dealers; ^c^ soft-drug dealers versus hard-drug dealers.

**Table 8:** Mental Health Problems Among Non-Dealers (*N* = 79), Soft-Drug Dealers (*N* = 63) and Hard-Drug Dealers (*N* = 83).

	Non-Dealers	Dealers			
	M (SD)	Soft M (SD)	Hard M (SD)	*F*(2, 222)	Cohen’s *d*
Anxiety problems	4.86 (3.43) ^a^	3.56 (2.76) ^a^	3.83 (2.92)	3.74 *	0.65
Depressive problems	6.68 (4.55)	5.83 (4.25)	5.99 (3.83)	0.88	0.13
Attention deficit/hyperactivity	5.54 (2.69)	5.62 (3.05)	5.88 (3.09)	0.29	0.22
Oppositional defiant problems	3.53 (2.15) ^b^	4.37 (2.22)	4.84 (2.06) ^b^	7.77 **	0.45

Note. Cohen’s *d* of 0.20 is a small effect, 0.50 a medium effect and 0.80 a large effect. * *p* < 0.05; ** *p* < 0.01; ^a^ non-dealers versus soft-drug dealers; ^b^ non-dealers versus hard-drug dealers.

## Data Availability

Data available on request due to privacy/ethical restrictions.

## Supplementary Materials

The following supporting information can be downloaded at: https://www.mdpi.com/article/10.3390/ijerph192416390/s1, STROBE Statement—Checklist of items that should be included in reports of *cross-sectional studies*.
